# Application of Visual Gene Clip-Based Tailored Therapy for the Eradication of *Helicobacter pylori*

**DOI:** 10.1155/2021/6150628

**Published:** 2021-04-10

**Authors:** Lili Yang, Ao Zou, Huihua Wu, Hai Guo, Fangting Zhang, Bing Zou, Junping Wang

**Affiliations:** Department of Gastroenterology, Peking University Shenzhen Hospital, Shenzhen, Guangdong Province, 518036, China

## Abstract

**Background:**

*Helicobacter pylori* eradication with therapies employing a proton pump inhibitor (PPI) and antimicrobial agents is mainly achieved via bacterial susceptibility to antimicrobial agents and the magnitude of acid secretion inhibition. However, annual eradication rates have greatly declined in Mainland China, and therefore, tailored *H. pylori* eradication regimens that inhibit acid secretion and employ optimal antimicrobial agents determined based on gene clip testing may improve eradication rates. This study was aimed at evaluating the efficacy of tailored *H. pylori* eradication therapy guided by visual gene clip testing for antibiotic resistance and PPI metabolism genotypes.

**Methods:**

This prospective study included 244 patients (141 men and 103 women aged 20–79 years) receiving initial treatment for *H. pylori* infection. Visual gene clip testing using gastric mucosal specimens was performed to detect antibiotic resistance to clarithromycin conferred by the A2142G and A2143G point mutations of the *H. pylori* 23S rRNA gene and to levofloxacin conferred by the Asn87 and Asp91 point mutations of the *H. pylori gyrA* gene. Patients received a 14-day bismuth quadruple therapy regimen guided by testing for antibiotic resistance and CYP2C19 polymorphisms, and primary *H. pylori* eradication was assessed at least 4 weeks after therapy.

**Results:**

*H. pylori* strains were successfully isolated from the gastric mucosa tissues of 244 patients. Antibiotic resistant isolates were identified in 63 patients, with clarithromycin resistance observed in 50 patients, levofloxacin resistance in 7 patients, and dual resistance in 6 patients. The PPI metabolic genotype of CYP2C19 was detected in 242 of 244 cases, and 97 cases were categorized as extensive metabolizers, 141 as intermediate metabolizers, and 4 as poor metabolizers. Among the 242 patients who received tailored therapy, the *H. pylori* eradication rate was 90.9% (95% confidence interval 87.3%~94.6%) in the intention-to-treat analysis and 96.9% (95% confidence interval 94.7%~99.2%) in the per protocol analysis.

**Conclusions:**

Tailored therapy for *H. pylori* infection guided by determination of antibiotic resistance and CYP2C19 polymorphism using visual gene chip technology may provide high clinical effectiveness as initial *H. pylori* eradication therapy.

## 1. Introduction

*Helicobacter pylori*, a gram-negative, spiral-shaped microaerophilic bacterium, is an important pathogen in gastrointestinal diseases, because *H. pylori* infection has been found to lead to asymptomatic chronic gastritis in 70% of the infected population, gastric ulcers in 15–20% of the infected population, dyspepsia in 10% of the infected population, and gastric cancer in 1% of the infected population [1–3]. In addition, numerous studies have implicated *H. pylori* in extragastric diseases, e.g., idiopathic thrombocytopenic purpura, iron-deficiency anemia, and vitamin B12 deficiency [4]. The World Health Organization classified *H. pylori* as a type 1 carcinogen in 1994 [5], and the Kyoto global consensus reported that *H. pylori*-related gastritis should be defined as an infectious disease [2]. The prevalence of *H. pylori* infection in China remains a significant health threat, affecting approximately 50% of the adult population [3]. Gastric cancer is the second most common cancer in China, with Chinese patients accounting for approximately 44% of the global gastric cancer incidence [6]. Thus, *H. pylori* eradication is crucial for cancer prevention in China. The *H. pylori* eradication rate with standard triple therapy, once considered the first-line treatment, has declined to below 80% in China [7], mainly due to the increased antibiotic resistance of *H. pylori* [8]. Proton pump inhibitors (PPIs) act synergistically with antibiotics to improve the efficacy *H. pylori* eradication regimens. PPIs are metabolized by cytochrome P450 enzymes, predominantly CYP2C19. Polymorphisms of CYP2C19 affect PPI metabolism and, subsequently, *H. pylori* eradication [9]. In the present study, we used visual gene clip technology to determine the antibiotic resistance of *H. pylori* to clarithromycin and levofloxacin as well as CYP2C19 gene polymorphisms to guide the initial treatment of *H. pylori* infection patients. Tailored therapies based on the results were provided, and eradication rates were evaluated.

## 2. Materials and Methods

This was a monocentric prospective clinical observational study conducted at Peking University Shenzhen Hospital, China.

### 2.1. Study Population

During the inclusion period of May 2015 to May 2017, consecutive patients who underwent upper endoscopy were enrolled in this study. Patients were eligible for recruitment if they were 24-78 years old and had documented *H. pylori* infection. The inclusion criteria were a diagnosis of *H. pylori* infection by a ^13^C-urea breath test and a rapid gastric mucosa urease test, endoscopically confirmed chronic erosive gastritis or peptic ulcer, and presentation for initial treatment. The exclusion criteria were previous eradication treatment for *H. pylori* infection; use of any drug that could influence the study results such as a PPI, H2-receptor blocker, bismuth, or antibiotics in the previous 4 weeks; gastrointestinal malignancy; previous gastric or esophageal surgery; severe concomitant disease; history of allergy to any of the study drugs; currently pregnant or lactating; and psychosis or severe neurosis. Of 250 patients who tested positive on a ^13^C-urea breath test, 6 were negative for a rapid urease test and were excluded from the study.

### 2.2. Study Design

The protocol was approved by the ethics committees of the hospital. The study was conducted according to the principles of the Declaration of Helsinki and the standards for Good Clinical Practice. All patients provided informed consent for participation in the study. With fully informed consent, the staff asked patients to provide general demographic data and medical history details, and specimens of gastric mucosa collected with a gastroscope were placed in sterile dry tubes and stored in −20°C until analysis. The specimens were used for tests of drug sensitivity and CYP2C19 genotype by gene clip testing.

### 2.3. Drug Resistance and CYP2C19 Polymorphism Testing

Gastric mucosal biopsies were obtained during esophagogastroduodenoscopy from all patients. Visual gene clip testing was performed with the *H. pylori* infection individualized Hp treatment detection kit (Ruikang Biotechnology, Shenzhen, China). The A2142G and A2143G point mutations of the *H. pylori* 23S rRNA gene, which confer antibiotic resistance to clarithromycin, and Asn87 and Asp91 point mutations of the *H. pylori gyrA* gene, which confer resistance to levofloxacin, were detected in the gastric mucosal specimens of all 244 patients. Additionally, genotyping was performed for the CYP2C19∗2 and CYP2C19∗3 polymorphisms to characterize PPI metabolism. Briefly, DNA extracted from gastric mucosal specimens was subjected to PCR amplification with biotin-labeled primers of target sequences. The resulting biotin-labeled amplicons were hybridized to DNA probes immobilized on aldehyde-coated glass slides and subsequently identified through combined tyramide signal amplification and silver amplification. Patients were classified into three groups by genotype based on the identification of the CYP2C19 wildtype (CYP2C19∗1) gene and the two mutated alleles (CYP2C19∗2 and CYP2C19∗3). Those without a mutation (∗1/∗1) were designated as the extensive metabolizer (EM) group, those with one mutation (∗1/∗2 or ∗1/∗3) were designated as the intermediate metabolizer (IM) group, and those with two mutations (∗2/∗2, ∗3/∗3, or ∗2/∗3) were designated as the poor metabolizer (PM) group. If any one mutation site was detected, resistance was assumed, and if two or more mutation sites were detected, double or multiple resistance was assumed.

### 2.4. Treatment Regimens

The tailored therapy (14 days) consisted of therapy drugs chosen according to antibiotic sensitivity and CYP2C19 genotyping results (Table 1). Colloidal bismuth pectin was used as the bismuth subsalicylate (BSS) component, and patients received 200 mg colloidal bismuth pectin twice daily.

### 2.5. Assessment of *H. pylori* Status

The primary outcome was *H. pylori* eradication confirmed by a ^13^C-urea breath test at 4 weeks posttherapy. Adverse events and compliance were monitored by telephone at 3, 7, and 14 days after the initiation of therapy. Patients were considered *H. pylori*-positive before treatment if they had a positive rapid urease test (*H. pylori* urease detection kit, Benxi Sitejie Biotechnology, Benxi, China) and ^13^C-urea breath test (^13^C-UBT) (UBT; UCBT Kit, Atom High Tech, Beijing, China). *H. pylori* eradication was assessed at least 4 weeks after the end of treatment by ^13^C-UBT. *H. pylori* eradication was considered successful if the UBT result was negative.

### 2.6. Statistical Analysis

Data are expressed as median (range) or percentage, as appropriate. All analyses were performed with SPSS statistical software (version 19.0, SPSS Inc., Chicago, IL). The primary efficacy variable was the eradication rate of *H. pylori*. Intention-to-treat (ITT) and per protocol (PP) statistical analyses were performed. The ITT analysis included all participants who had taken at least one study medication. Patients who did not return to the final follow-up visit and for whom the result of the posttreatment testing was unavailable within 6 months after the prescription of therapy were considered lost to follow-up and, thus, treatment failures. PP analysis included all patients who took at least 90% of the study medication and underwent the final follow-up, including obtaining the result of the posttreatment testing. We calculated the mean values with standard deviation (SD) for continuous variables and percentages with 95% confidence intervals (CI) for categorical variables.

## 3. Results

Of the 244 enrolled patients, 141 (57.8%) were men and 103 (42.2%) were women. The median age was 48.5 years (range, 24–78 years), and no significant difference in age was observed between men and women.

Table 2 summarizes the results of genotypic antibiotic resistance testing. Of the 244 patients tested, 181 (74.18%) were susceptible to both clarithromycin and levofloxacin. Of the remaining 63 patients, 50 and 7 patients were resistant to clarithromycin and levofloxacin, respectively. Six patients had dual resistance.

The CYP2C19 genotyping analysis results are reported in Table 3. Of the 242 patients with CYP2C19 genotyping results, the largest percentage were IMs (141, 58.26%), followed by EMs (97, 40.08%) and PMs (4, 1.66%). CYP2C19 polymorphisms were not detected in two patients.

Both CYP2C19 genotypes were detected in 242 cases, and drug resistance was detected in 244 patients. Of the 242 patients, 6 patients were lost to follow-up, 2 patients withdrew from the study due to intolerable adverse effects (major symptoms included nausea, vomiting, anorexia, dizziness, and diarrhea), 7 patients showed poor compliance, 7 patients experienced eradication failure, and 220 patients achieved successful eradication. The initial eradication rate of *H. pylori* was 90.9% by ITT analysis (95% CI 87.3%~94.6%) and 96.9% by PP analysis (95% CI 94.7%~99.2%). The eradication rates of *H. pylori* in CYP2C19 EM, IM, and PM carriers were 88.66%, 92.20%, and 100%, respectively, in the ITT analysis, showing no statistical difference; in the PP analysis, the rates were 95.56%, 97.74%, and 100%, respectively (Table 4).

## 4. Discussion

*H. pylori* infection is a public health problem worldwide that affects approximately 50% of global populations. The incidence of *H. pylori* infection in China is high and continues to increase each year [10]. All regimens for *H. pylori* eradication therapy are drug combinations, including standard triple therapy, bismuth quadruple therapy, nonbismuth quadruple therapy, sequential therapy, and concomitant therapy [11]. With the increasing frequency of antibiotic resistance, the *H. pylori* eradication rate with these therapies has declined to as low as 80% [12]. Moreover, a meta-analysis showed that the eradication rate with standard triple therapy is only 70% in China [13]. According to the performance assessment criteria described by Graham et al. [14], the efficacy of *H. pylori* therapy can be categorized based on an ITT analysis of eradication rates into excellent (>95%), good (90–95%), fair (85–89%), poor (81–84%), and unacceptable (≤80%). The Kyoto global consensus report on *H. pylori* gastritis emphasized that eradication regimens should be tailored and based on local profiles of antibiotic resistance and genotypes of drug-metabolizing enzymes [2]. Gastroscopy is popular in China and facilitates tailored treatment based on gastroscopic findings. The present study detected both antibiotic resistance and CYP2C19 gene polymorphisms using gene chip technology in gastric mucosal samples of patients awaiting initial treatment for *H. pylori* infection, and the obtained information was used to guide tailored treatment and evaluate the clinical value of this approach.

The antibiotics most commonly used for *H. pylori* eradication include amoxicillin, tetracycline, furazolidone, clarithromycin, metronidazole, and levofloxacin. A meta-analysis showed that the primary resistance rates of *H. pylori* to amoxicillin, tetracycline, and furazolidone in China are 3.1%, 3.9%, and 1.7%, respectively, which are considered relatively low and reflective of a stable status. By comparison, the primary resistance rates to clarithromycin, metronidazole, and levofloxacin in China are 28.9%, 63.8%, and 28.0%, respectively, which are considered high and show continued increases each year [8, 15–17]. Moreover, resistance to multiple drugs also occurs [8, 15–17]. The resistance rate to metronidazole exceeds 90% in southern China. Thus, metronidazole is not suitable for eradication therapy in the southern city, Shenzhen, and resistance to clarithromycin and levofloxacin should be considered during the process of eradication treatment [18]. In the present study, 22.9% of patients with *H. pylori* infection were found to have primary clarithromycin resistance, consistent with the average prevalence worldwide. Notably, the Fifth Chinese National Consensus Report on the management of *H. pylori* infection recommended that antibiotic susceptibility testing be performed prior to the use of clarithromycin-based triple therapy in populations with a prevalence of clarithromycin resistance exceeding 15% [19]. Therefore, our findings indicate the need for antibiotic resistance testing prior to the administration of clarithromycin-based eradication therapy in patients in Shenzhen, Guangdong, and if antibiotic resistance testing is not available, clarithromycin as an empirical treatment option should be avoided in this region. In this study, the prevalence of primary levofloxacin resistance was only 5.3%, which was much lower than the rate of 18% reported previously in the Asia-Pacific region [15]. Levofloxacin-based therapy is therefore recommended as the first-line choice for patients with *H. pylori* infection in this region.

Adjunct administration of PPIs with antibiotics improves eradication rates [17]. The primary mechanism of action is thought to be the suppression of gastric acid secretion, which consequently reduces the degradation of antibiotics, thus enhancing their concentrations and antimicrobial activity in the gastric microenvironment [20**]**. Members of the CYP450 family, including CYP2C19 and CYP3A4, are the principal enzymes responsible for the hepatic metabolism of PPIs. CYP2C19 in particular plays a pivotal role, given that the genotype of CYP2C19 determines the speed at which certain PPIs are metabolized. Polymorphism of CYP2C19 is related to heredity, race, individual, and region [21]. Among the Asian population, the most common CYP2C19 genotypes are the EM and IM types, with the PM type found in few patients [22]. PPIs are metabolized by CYP2C19 at different rates [17]. Rabeprazole is metabolized primarily via a nonenzymatic process and thus is not greatly influenced by CYP2C19 polymorphisms [21]. Tailored treatment with adjustment of the medication regimen or dosage according to predicted pharmacokinetics determined by detecting the CYP2C19 genotype can improve the eradication rate [22, 23]. Our data showed that the prevalence rates of the EM and IM types were 40.08% and 58.26%, respectively, while that of the PM type was only 1.66% in our patients. Therefore, rabeprazole should be the first-line treatment for patients in this region, and the PPIs affected by the CYP2C19 genotype should be appropriately increased in dose or the frequency of administration to ensure the acid suppression effect [24]. It should be noted that the prevalence rate of the PM type in our patients was lower than that in patients with gastrointestinal diseases of Han Chinese ethnicity in north China (12.0%) [25] but comparable to the rates observed in white and black populations in a previous study (2–6% and 1%, respectively) [26]. The reason for this discrepancy in the CYP2C19 genotype between our patients and other Chinese populations is unknown. Given the considerable geographic and ethnicity disparities in the CYP2C19 genotype, one possible explanation may be related to the mixed ethnic background of patients from Shenzhen, many of whom have immigrated to the city from different regions of China. There is also a possibility that the discrepancy may be due to the limited sample size, and further investigation is warranted.

A previous study reported an *H. pylori* eradication rate of 88.7% in patients who received tailored therapy, a rate that is significantly higher than that in patients who receive empiric bismuth quadruple or concomitant therapy [27]. A comparison study of tailored treatment with bismuth quadruple, concomitant, sequential, and triple therapy in China showed that tailored treatment offered advantages in terms of safety, compliance, and eradication rate [28]. In the present study, we designed tailored bismuth quadruple therapy regimens based on antibiotic resistance and CYP2C19 genotypes. This approach achieved eradication rates of 90.9% (95% CI, 87.3%–94.6%) in the ITT analysis and 96.9% (95% CI is 94.7%–99.2%) in the PP analysis, which falls within the ideal range for eradication rates (>90%). The eradication rate in CYP2C19 PM carriers was the highest, but it did not differ significantly from the rates in CYP2C19 EM and IM carriers. Furthermore, the tailored treatment approach can avoid the extra costs of repeated treatment and treatment failure caused by drug resistance. Therefore, the presented approach to tailored treatment warrants further clinical verification and promotion in clinical practice.

Gene chip technology has emerged over the last decade as a potential tool for antibiotic resistance monitoring, given its capacity for screening a wide diversity of genes. Gene chip technology offers greater sensitivity and accuracy than bacterial culture for detecting *H. pylori* infection and assessing antibiotic resistance [29]. Gene chip technology shares the same accuracy and sensitivity as gene sequencing technology, but because it is simpler and more efficient and requires only portable equipment, the cost is greatly reduced and is generally lower than the cost of conventional antibiotic susceptibility testing methods [30].

## 5. Conclusions

In this study, gene chip technology was used to guide the tailored clinical treatment for *H. pylori* eradication, and satisfactory therapeutic effects were observed. With the development and advancement of gene science, tailored treatment for *H. pylori* eradication based on this technology may be applied widely in clinical practice. Because this study was a single-center, nonrandomized controlled observation study with a limited sample size, further multicenter studies with large sample sizes are needed to confirm the findings of the present study.

## Figures and Tables

**Table 1:** Antibiotics and proton pump inhibitors used in personalized *H. pylori* eradication therapy regimens.

Antibiotic resistance		Dosing and frequency (2 weeks)				Metabolism phenotype	Dosing and frequency (2 weeks)	
Clarithromycin	Levofloxacin	Clarithromycin	Levofloxacin	Furazolidone	Amoxicillin		Rabeprazole	Pantoprazole
Susceptible	Susceptible	0.5 bid			1.0 bid	EM or IM	10 mg bid	
						PM		40 mg bid
Susceptible	Resistant	0.5 bid			1.0 bid	EM or IM	10 mg bid	
						PM		40 mg bid
Resistant	Susceptible		0.5 qd		1.0 bid	EM or IM	10 mg bid	
						PM		40 mg bid
Resistant	Resistant			0.1 bid	1.0 bid	EM or IM	10 mg bid	
						PM		40 mg bid

**Table 2:** Genotypic results for determining antibiotic resistance in patients with *H. pylori* infection.

Strain type	Number	Mutations in gyrA		Mutations in 23S rRNA	
		Asn87	Asp91	A2142G	A2143G
Simple mutation	18			A2142G	
	32				A2143G
	5	Asn87			
	2		Asp91		

Dual mutation	3	Asn87			A2143G
	2	Asn87		A2142G	
	1		Asp91		A2143G

**Table 3:** Genotypic and phenotypic results for the CYP2C19 gene in patients with *H. pylori* infection.

Phenotype	Drug metabolic rate	Genotypes	*n* (%)
EM	Extensive	∗1/∗1	97 (40.08)

IM	Intermediate	∗1/∗2; ∗1/∗3	141 (58.26)

PM	Poor	∗2/∗2; ∗2/∗3; ∗3/∗3	4 (1.66)

EM: extensive metabolizer; IM: intermediate metabolizer; PM: poor metabolizer.

**Table 4:** *H. pylori* eradication rates by CYP2C19 phenotype.

	Eradication rate, *n*/*N* (%)	95% confidence interval	*p*
Intent to treatment			
CYP2C19 EM type	86/97 (88.66)	82.24–95.08	>0.05
CYP2C19 IM type	130/141 (92.20)	87.72–96.68	
CYP2C19 PM type	4/4 (100)	100–100	
Per protocol			
CYP2C19 EM type	86/90 (95.56)	91.22–99.90	>0.05
CYP2C19 IM type	130/133 (97.74)	95.188–100	
CYP2C19 PM type	4/4 (100)	100–100	

## Data Availability

The data sets generated and analyzed during the current study are available from the corresponding author on reasonable request.
